# Influence of Different Restoring Materials on Stress Distribution in Prosthesis on Implants: A Review of Finite Element Studies

**DOI:** 10.1055/s-0042-1747955

**Published:** 2022-06-21

**Authors:** Fabiano Resmer Vieira, Sandro Basso Bitencourt, Cleber Davi Del Rei Daltro Rosa, André Bueno Vieira, Daniela Micheline dos Santos, Marcelo Coelho Goiato

**Affiliations:** 1 Department of Dental Materials and Prosthodontics, School of Dentistry, Aracatuba, São Paulo State University, Aracatuba, Brazil; 2 Department of Dentistry, University Center of Espírito Santo-UNESC, Colatina, Brazil; 3 Department of Dental Materials and Prosthodontics, School of Dentistry, Aracatuba, São Paulo State University, Aracatuba, Brazil; 4 Undergraduate Student , Department of Dentistry at the State University of Maringá, Brazil; 5 Department of Dental Materials and Prosthodontics, School of Dentistry, Aracatuba, São Paulo State University, Aracatuba, Brazil

**Keywords:** dental implant, finite element analysis, ceramics, occlusal surface

## Abstract

The selection of material used on the occlusal surface of implant-supported prostheses is important, as these materials can transmit destructive forces to the interface between the alveolar bone and the implant. Different prosthetic materials are suggested for implant-supported prostheses. The choice of prosthetic material is a controversial issue, and there is a consensus that implant survival is not affected by the prosthetic material. Three-dimensional finite element studies are often used in dentistry to estimate the stress distribution that occurs in the implant system, peri-implant bone, and prosthetic components. To analyze the influence of the prosthetic restorative material on the stresses in bone tissue and peri-implant through a literature review of three-dimensional finite element studies. The search for articles was performed in the PubMed/Medline database up to November 2021. The selected articles were independently evaluated by two different reviewers. The information collected was author and year of publication, dimensions of implants used, the material used in the prosthetic crown, simulated force and direction, and conclusion and effect. After searching, 14 studies were selected for full reading, and based on the inclusion and exclusion criteria, all could be included in this review. The articles were based on evidence-based laboratory medicine. After analyzing these articles, it was concluded that the prosthetic materials used on the occlusal surface do not interfere with the destruction of stresses to the bone and peri-implant tissue, both in single prostheses and protocol-type prostheses, when three-dimensional finite element method is used.

## Introduction

Dental implants have improved the quality of life of millions of patients in recent decades and have shown a high predictability of success. 1 The high success rates and long-term follow-up (over 20 years) of patients treated with osseointegrated dental implants have attracted the interest of clinicians and researchers worldwide. 2 Occlusal loading of osseointegrated implants is a determining factor in the longevity of treatments with implants. The selection of material used on the occlusal surface of implant-supported prostheses is also important, as these materials can transmit damaging forces to the interface between the alveolar bone and the implant. 3

Different prosthetic materials are suggested for the fabrication of implant-supported prostheses. The choice of this material is controversial although there is a consensus that implant survival is not affected by the prosthetic material. 4 Skalak et al 5 stated the theory that loading an implant made of a hard occlusal material, either porcelain or metal, can result in high-intensity loading between the implant and the supporting bone. While a material with a low modulus of elasticity has stress-absorbing properties, it can prevent the surrounding bone from possible destruction linked to the magnitude of the load. 6

Three-dimensional finite element analyses (3D-FEA) are frequently used in dentistry to estimate the stress distribution that occurs in the implant system, peri-implant bone, and prosthetic components. 6 3D-FEA allows the simulation of a condition that would be impossible to achieve in a clinical study. Therefore, traditional finite elements have become a useful tool to study stress distribution in implant dentistry. 7

The purpose of this review of the literature on finite element studies is to investigate the influence of the prosthetic material on the stresses induced in bone tissue in implant-supported prostheses.

## Methods

The search for articles of this review of the literature was performed in the PubMed/Medline database for articles published up to November 2021. The search strategy used was (finite element analysis) AND (occlusal device OR occlusal surface OR occlusal materials OR veneering materials) AND (implants OR dental implants). The inclusion criteria were studies using the 3D-FEA methodology that evaluated the stress distribution in bone tissue, among different prosthetic/restorative materials, published only in English. The exclusion criteria were studies that did not follow the 3D-FEA methodology. The selected articles were independently evaluated by two different reviewers. The information collected was author and year of publication, dimensions of implants used, the material used in the prosthetic crown, simulated force and direction, and conclusion and effect.

Articles of the *in vitro* study were selected following evidence-based laboratory medicine. 8 These principles are (1) asking the question, (2) searching for evidence, (3) appraising the evidence, (4) applying the evidence, and (5) assessing the experience.

## Results

**Fig. 1:**
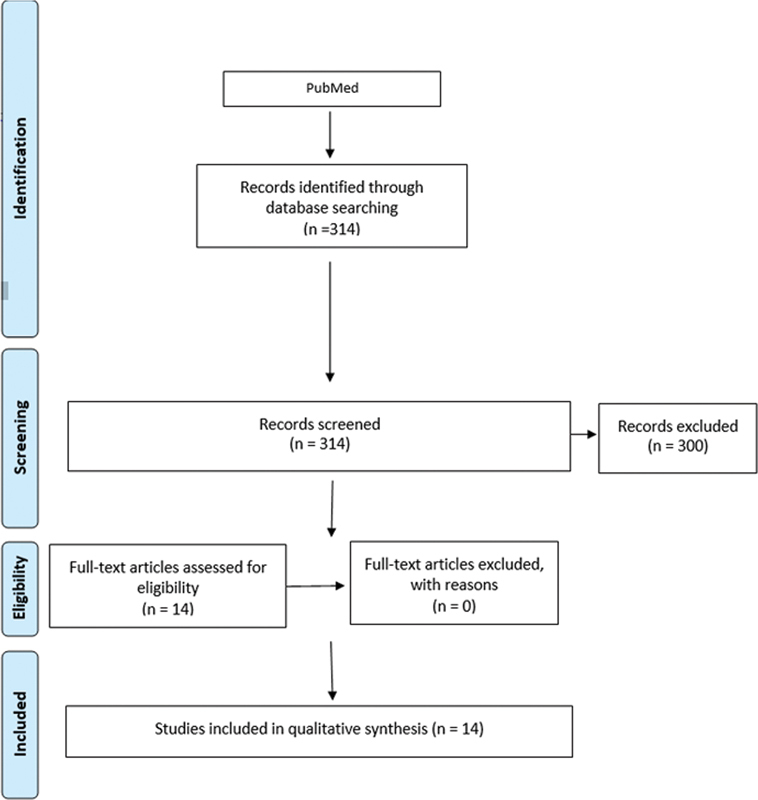
Details of the search strategy performed.

During the search process, 314 references were found, of which 14 were selected after reading the title and abstract, to be analyzed for their full-text. After this step, all 14 articles were included for data collection. The search strategy is detailed in Fig. 1 . The individual details of the studies included in this review can be seen in Table 1 .

**Table 1:** Characteristics of the studies included in this review

Author	Implant information	Prosthetic crown	Simulated force	Outcome	Effect
Yegin and Atala, 2020 14	Internal connection at the level 4.1 mm × 12 mm	**Lower first molar** 1. Monolithic LD 2. LD as a veneering ceramic 3. LD reinforced with monolithic zirconia 4. Reinforced LD as a veneering ceramic	300 N Axial	Von Mises stresses were relatively similar and concentrated in the coronal part of implants and abutments in all groups. The different restorative materials did not influence the stress distribution although monolithic crowns reduced the stress concentration on the implant and the bone.	Negative
Mendes Tribst et al, 2019 13	Internal hexagon 3.75 mm × 11 mm	Molar 1. Monolithic zirconia 2. Monolithic LD 3. Monolithic Hybrid Ceramics	300 N Oblique (30°)	There were no differences between zirconia, LD, and hybrid ceramic crowns in the distribution of forces in the bone. The use of the combination of a crown with a low modulus of elasticity and a mesostructure with a high modulus of elasticity on Ti-base demonstrated a better dissipation of forces.	Negative
Sotto-Maior et al, 2012 12	External hexagon 5 mm × 7 mm	Lower first molar 1. PFM (Gold IV infrastructure and feldspathic ceramic veneer) 2. All-ceramic (zirconia framework and feldspathic ceramic veneer)	200 N Axial Oblique (45°)	The dissipation of forces was not influenced by the prosthetic material, the occlusal force is the factor that has the greatest weight concerning the stresses generated in the implants, the second most important being the abutment.	Negative
Sevimay et al, 2005 2	4.1 mm × 10 mm	Lower second premolar 1. IPS Empress 2 2. In-Ceram 3. PFM with cobalt-chromium 4. PFM with gold-silver-palladium	300 N Axial	Different materials did not influence the distribution of forces in the bone and peri-implant bone tissue. However, in the abutment and crown structure, stress distributions and locations were affected by the stiffness of the material, because materials with the highest modulus of elasticity concentrated higher stress.	Negative
Sanninno et al, 2010 17	4.5 mm × 13 mm	1. Zirconia 2. Micro-hybrid composite	30 N 60 N 120 N 240 N 500 N 600 N 800 N Axial and oblique (45°)	In axial loading, the use of zirconia in the abutment and crown allows for less tension (∼5–10%) than the other combinations of materials investigated, in the bone region around the implant neck. In oblique loads, the use of titanium abutment and a micro-hybrid crown composite allows for the transfer of occlusal loads more uniformly and with slightly lower peak von Mises stress values (7–11%) than in the other cases. However, this material choice, as well as the combination of zirconia abutment and micro-hybrid composite in the crown, produces the greatest stress gradients in the cement layer.	Positive
Juodzbalys et al, 2005 11	3.8 mm × 12 mm	Lower first molar 1. Vita VMK 68 Seer 2. GC GRADIA	143 N - 0° 500 N - 90° 1000 N - 120°	The use of different materials had less influence on the stresses in the supporting bone with 1% of the variance.	Negative
Junior et al, 2013 20	External hexagon 3.75 mm × 10 mm and 5 mm × 10 mm	Lower first molar 1. Nickel-chrome metallic crown 2. Nickel-chromium framework with feldspathic ceramic veneer 3. Nickel-chrome framework with resin composite veneer 4. Nickel-chrome framework with acrylic resin veneer	200 N Axial 100 N Oblique	There was no significant difference in the distribution of stresses in the bone to the different veneering materials.	Negative
Gungor and Yilmaz, 2014 4	4 mm × 11.5 mm	Fixed partial prosthesis (central incisor, lateral incisor, and canine) 1. LD 2. Zirconia	76.5 N Axial 534 N Oblique (30°)	Different prosthetic materials did not change the pattern of stress distribution in the bone. Stresses that occurred in the trabecular bone were similar between the models under both loadings.	Negative
Mourya et al, 2021 18	Internal connection 4.2 mm × 10 mm	Upper premolar 1. PFM 2. PEEK	1000 N Axial 500N Oblique (30°)	PEEK crowns with a composite resin layer in both loadings produced less stress on the bone and implant, but stresses on the abutment were lower in the PFM group.	Positive
Ercal et al, 2021 6	4.1 mm × 7 mm	Lower premolar 1. PFM with cobalt-chromium 2. All ceramic on a zirconia framework 3. Monolithic zirconia 4. Composite resin on a zirconia framework	200 N Axial 100 N Oblique (30°)	There were no differences in von Mises stresses around implants between the different materials used in prosthetic crowns.	Negative
Papavasiliou et al, 1996 9	4 mm × 11 mm	Lower canine 1. Gold coated with acrylic resin 2. PFM	200 N Axial Oblique	Changing the prosthesis veneering material had no significant effect on stress levels or distribution at the bone-implant interface.	Negative
Ferreira et al, 2021 15	4 implants External hexagon 3.75 mm X 11 mm	Lower protocol 1. Acrylic resin 2. Porcelain	100 N Oblique (30°)	Although resin teeth had lowered von Misses stress values, groups with porcelain teeth significantly decreased stresses on metallic frameworks. Porcelain-induced stresses did not differ from resin when evaluating implants and bone.	Negative
Sertgoz et al, 1997 10	6 implants 4 mm × 12 mm	Lower protocol 1. Acrylic resin 2. Composite resin 3. Porcelain	172 N Axial	Maximum stresses within cortical and cancellous bone did not differ as a result of using different materials on the occlusal surface.	Negative
Lemos et al, 2021 16	4 mm × 10 mm	1. PFM 2. Monolithic zirconia	200 N Axial 100 N Oblique	No differences were observed between PFM and monolithic zirconia crowns in terms of microstrain and stress distribution in cortical bone, implants, or abutments.	Negative

Abbreviations: LD, lithium disilicate; N, Newtons; PFM, porcelain fused to metal; PEEK, polyetheretherketone.

The selected studies were dated from 1996 to 2021. The simulated implants were of varying diameters, ranging from 3.8 mm to 5 mm. The length of the implants also varied, with the shortest length being 7 mm and the longest being 13 mm. Regarding the prosthetic materials, a variety of metal-ceramic prostheses can be seen, varying the material of the infrastructure and the veneering ceramic, in addition, prostheses in lithium disilicate and zirconia, acrylic resin, and composite resin. The simulated forces ranged from 30 to 1000 N, using either axial or oblique loads.

Twelve studies found no differences in force dissipation in bone tissue between different prosthetic materials 2 4 6 9 10 11 12 13 14 15 16 20 . Only two studies found a positive relationship between the restorative material and bone tissue tension 17 18 .

## Discussion

Biomechanical considerations are recognized as being one of the most important factors for the long-term success of osseointegrated implants. Among the methods for evaluating implant biomechanics, 3D-FEA has been widely used for the quantitative assessment of bone stresses. 2 This analysis identifies stresses and their dissipation at the prosthesis-implant-bone interface, which can be difficult to assess by other biomechanical methods. 14

After the review of included studies, it was found that most of the articles indicate that the prosthetic material does not influence the generation of tension and dissipation in the bone and peri-implant tissue. This can be justified due to the use of different prosthetic materials having less influence on the stresses in the supporting bone with 1% of the variance. 11 According to Sotto-Maior et al, 12 the dissipation of forces was not influenced by the prosthetic material although the occlusal force is the factor that has the highest weight about the stresses generated in the implants, abutment being the second. Despite not affecting bone tissue, occlusal materials show differences in stress distributions in the crown structure and abutment.

Sevimay et al 2 evaluated prosthetic crowns made with IPS Empress 2, In-Ceram, PFM with a chromium-cobalt framework, PFM with a gold-silver-palladium framework, and states that the different prosthetic materials did not influence the distribution of forces in bone and peri-implant bone tissue. However, when evaluating the ceramic, IPS Empress 2 showed the highest stress concentration. When the stress distribution in the framework was evaluated, the stress values were different for each model. In-Ceram porcelain (173 MPa) and PFM crown with a cobalt-chromium framework (149 MPa) induced higher von Mises stress values than PFM crown with a gold-silver-palladium framework (108 MPa) and IPS Empress 2 (119 MPa). The reason for these differences may be related to the elastic modulus of the materials. In-Ceram and PFM crown with chromium-cobalt framework have a higher modulus of elasticity compared with IPS Empress 2 and PFM crown with a gold-silver-palladium framework.

Sannino et al 17 analyzed the stresses of the prosthesis-implant system and showed that the choice of material was crucial for the distribution of stresses in different components. It was noted that due to the large difference in the hardness between the materials of the system, the main stress gradient in the cement layer increased in the situation of zirconia abutment with micro-hybrid composite core and titanium abutment with a micro-hybrid composite core. For the situation of zirconia on abutment and core, and titanium abutment and zirconia core, the stress distribution in the cement layer was more homogeneous. Higher failure risks for the cement layer placed between the core and the abutments were found when a micro-hybrid composite core was used.

Gungor and Yilmaz 4 reported that higher stress levels were observed in the models with zirconia (93.6 MPa) compared with models with lithium disilicate (76.3 MPa). One justification for this is that stresses in the framework materials increased with the decrease in the modulus of elasticity of the layering material. Higher differences between the modulus of elasticity of the framework and the veneer material transmit greater concentrations of stress in the framework. 19 Yegin and Atala 14 also agree that higher differences between the modulus of elasticity of the infrastructure and the veneer ceramics lead to a higher concentration of stress in the framework. Thus, the monolithic crowns showed a decrease in stress concentration, as the stresses were more concentrated on the ceramic surface due to the elastic modulus being the same throughout the prosthesis, which reduced the load transmission to the implant and the bone, consequently.

The study by Mourya et al 18 reveals that the use of a material with a lower modulus of elasticity in the crown, such as PEEK crowns with a composite resin layer, implies greater stress on the abutment than a metal-ceramic crown. The PEEK group in the axial loading presents 514 MPa in the abutment, While the metal-fused porcelain crown has a tension of 123Mpa. In the oblique loading, which is the most harmful to the implants, these values increase to 1347 MPa (PEEK) and 400 MPa (PFM). Due to this, the use of prosthetic materials with a low modulus of elasticity may be associated with failures in the abutment region, with the retaining screw being the most subject to failure. Alves Gomes et al 3 observed that porcelain crowns absorbed less stress than composite resin crowns. The use of porcelain as a veneer material reduced the stress that was transmitted to the retaining screw. Composite resin has a low modulus of elasticity and is more deformable than porcelain. Thus, resin exhibits greater displacement and transfers stress directly to the retaining screw, different from the porcelain. Low abrasion resistance is a disadvantage of the composite resin. If the occlusal scheme and morphology cannot be maintained over time, undesirable lateral forces may increase.

When evaluating complete dentures fixed to implants, studies 10 20 agreed that different occlusal materials did not influence the tension transmitted to the bone tissue. However, Ferreira et al 20 shows that although the resin teeth had lower values of von Mises stress, the groups with porcelain teeth significantly decreased the stresses on the metallic frameworks.

Although different occlusal materials do not influence the tension transmitted to the bone and peri-implant tissue, there is a tendency where materials with low elastic modulus transmit greater tensions to the infrastructure materials both in single prostheses and in protocol-type prostheses. In contrast to the 12 studies that did not show a positive relationship between the prosthetic material and the increase in stress in the bone tissue, two articles found a positive result, which can be explained by the different designs of the 3D-FEA studies, where the change in the implant geometry and bone density between studies may explain the discrepancies between the results. Finite element studies allow an approximation of the behavior of the material to the real situation. 12

Therefore, further investigations related to dynamic applications of forces and long-term clinical studies are needed to assist the dentist in choosing the appropriate prosthetic material in implant-supported restorations.

## Conclusion

Evaluating the stress distribution by 3D-FEA, the prosthetic materials used on the occlusal surface did not interfere with the distribution of stresses to the bone and peri-implant tissue, both in single prostheses and protocol-type prostheses.

## References

[1] Santiago Junior J F Verri F R Almeida D A de Souza Batista V E Lemos C AA Pellizzer E P Finite element analysis on influence of implant surface treatments, connection and bone types Mater Sci Eng C 2016 63 292 300 10.1016/j.msec.2016.02.061 27040222

[2] Sevimay M Usumez A Eskitascioglu G The influence of various occlusal materials on stresses transferred to implant-supported prostheses and supporting bone: a three-dimensional finite-element study J Biomed Mater Res B Appl Biomater 2005 73 01 140 147 15742379 10.1002/jbm.b.30191

[3] Gomes ÉA Barão V A Rocha E P de Almeida ÉO Assunção W G Effect of metal-ceramic or all-ceramic superstructure materials on stress distribution in a single implant-supported prosthesis: three-dimensional finite element analysis Int J Oral Maxillofac Implants 2011 26 06 1202 1209 22167424

[4] Bankoğlu Güngör M Yılmaz H Evaluation of stress distributions occurring on zirconia and titanium implant-supported prostheses: a three-dimensional finite element analysis J Prosthet Dent 2016 116 03 346 355 27063944 10.1016/j.prosdent.2016.01.022

[5] Skalak R Biomechanical considerations in osseointegrated prostheses J Prosthet Dent 1983 49 06 843 848 6576140 10.1016/0022-3913(83)90361-x

[6] Ercal P Taysi A E Ayvalioglu D C Eren M M Sismanoglu S Impact of peri-implant bone resorption, prosthetic materials, and crown to implant ratio on the stress distribution of short implants: a finite element analysis Med Biol Eng Comput 2021 59 04 813 824 33728596 10.1007/s11517-021-02342-w

[7] de Souza Batista V E Verri F R Almeida D AF Santiago Junior J F Lemos C AA Pellizzer E P Evaluation of the effect of an offset implant configuration in the posterior maxilla with external hexagon implant platform: a 3-dimensional finite element analysis J Prosthet Dent 2017 118 03 363 371 28222876 10.1016/j.prosdent.2016.10.033

[8] Christenson R H Snyder S R Shaw C S Laboratory medicine best practices: systematic evidence review and evaluation methods for quality improvement Clin Chem 2011 57 06 816 825 21515742 10.1373/clinchem.2010.157131

[9] Papavasiliou G Kamposiora P Bayne S C Felton D A Three-dimensional finite element analysis of stress-distribution around single tooth implants as a function of bony support, prosthesis type, and loading during function J Prosthet Dent 1996 76 06 633 640 8957790 10.1016/s0022-3913(96)90442-4

[10] Sertgöz A Finite element analysis study of the effect of superstructure material on stress distribution in an implant-supported fixed prosthesis Int J Prosthodont 1997 10 01 19 27 9484066

[11] Juodzbalys G Kubilius R Eidukynas V Raustia A M Stress distribution in bone: single-unit implant prostheses veneered with porcelain or a new composite material Implant Dent 2005 14 02 166 175 15968189 10.1097/01.id.0000165030.59555.2c

[12] Sotto-Maior B S Senna P M da Silva W J Rocha E P Del Bel Cury A A Influence of crown-to-implant ratio, retention system, restorative material, and occlusal loading on stress concentrations in single short implants Int J Oral Maxillofac Implants 2012 27 03 e13 e18 22616067

[13] Tribst J PM Dal Piva A MO Borges A LS Bottino M A Different combinations of CAD/CAM materials on the biomechanical behavior of a two-piece prosthetic solution Int J Comput Dent 2019 22 02 171 176 31134223

[14] Yeğin, Elif, Mustafa Hayati Atala.Comparison of CAD/CAM manufactured implant-supported crowns with different analyses Int J Implant Dentistry 2020 6 01 1 11 10.1186/s40729-020-00267-x PMC7588579 33106916

[15] Ferreira M B Barão V A Faverani L P Hipólito A C Assunção W G The role of superstructure material on the stress distribution in mandibular full-arch implant-supported fixed dentures. A CT-based 3D-FEA Mater Sci Eng C 2014 35 92 99 10.1016/j.msec.2013.10.022 24411356

[16] Lemos C AA Verri F R Noritomi P Y Souza Batista V E Cruz R S de Luna Gomes J M Pellizzer E P Biomechanical evaluation of different implant–abutment connections, retention systems, and restorative materials in the implant-supported single crowns using 3D finite element J Oral Implantol 2021 10.1563/aaid-joi-D-20-00328 34091686

[17] Sannino G Marra G Feo L Barlattani A 3D finite element non linear analysis on the stress state at bone-implant interface in dental osteointegrated implants Oral Implantol (Rome) 2010 3 03 26 37 23285387 PMC3399188

[18] Mourya A Nahar R Mishra S K Chowdhary R Stress distribution around different abutments on titanium and CFR-PEEK implant with different prosthetic crowns under parafunctional loading: a 3D FEA study J Oral Biol Craniofac Res 2021 11 02 313 320 33816100 10.1016/j.jobcr.2021.03.005 PMC8005827

[19] Möllers K Pätzold W Parkot D Influence of connector design and material composition and veneering on the stress distribution of all-ceramic fixed dental prostheses: a finite element study Dent Mater 2011 27 08 e171 e175 21592550 10.1016/j.dental.2011.04.009

[20] Santiago Junior J F Pellizzer E P Verri F R de Carvalho P S Stress analysis in bone tissue around single implants with different diameters and veneering materials: a 3-D finite element study Mater Sci Eng C 2013 33 08 4700 4714 10.1016/j.msec.2013.07.027 24094178

